# Synchronized human skeletal myotubes of lean, obese and type 2 diabetic patients maintain circadian oscillation of clock genes

**DOI:** 10.1038/srep35047

**Published:** 2016-10-19

**Authors:** Jan Hansen, Silvie Timmers, Esther Moonen-Kornips, Helene Duez, Bart Staels, Matthijs K. C. Hesselink, Patrick Schrauwen

**Affiliations:** 1 Department of Human Biology and Human Movement Sciences, NUTRIM School for Nutrition and Translational Research in Metabolism, Maastricht University Medical Center, Maastricht, The Netherlands; 2 Institut Pasteur de Lille, Lille, France; 3 Institut National de la Santé et de la Recherche Médicale Unité Mixte de Recherche 1011, Lille, France; 4 Univ. Lille, European Genomic Institute for Diabetes, Lille, France

## Abstract

Cell and animal studies have demonstrated that circadian rhythm is governed by autonomous rhythmicity of clock genes. Although disturbances in circadian rhythm have been implicated in metabolic disease development, it remains unknown whether muscle circadian rhythm is altered in human models of type 2 diabetes. Here we used human primary myotubes (HPM) to investigate if rhythmicity of clock- and metabolic gene expression is altered in donors with obesity or type 2 diabetes compared to metabolically healthy donors. HPM were obtained from skeletal muscle biopsies of four groups: type 2 diabetic patients and their BMI- and age-matched obese controls and from lean, healthy and young endurance trained athletes and their age-matched sedentary controls. HPM were differentiated for 7 days before synchronization by serum shock followed by gene expression profiling over the next 72 hours. HPM display robust circadian rhythms in clock genes, but REVERBA displayed dampened rhythmicity in type 2 diabetes. Furthermore, rhythmicity in NAMPT and SIRT1 expression was only observed in HPM from trained athletes. Rhythmicity in expression of key-regulators of carbohydrate and lipid metabolism was modest. We demonstrate that in human skeletal muscle REVERBA/B, NAMPT and SIRT1 circadian rhythms are affected in donors of sedentary life style and poor health status.

Skeletal muscle plays an important role in maintaining whole body energy and substrate metabolism and is responsible for ~80% of postprandial glucose uptake in humans^1^. Type 2 diabetes (T2D) is indeed characterized by disturbances in skeletal muscle insulin sensitivity and mitochondrial function^2^,^3^, and the disability of skeletal muscle to adjust substrate oxidation to glucose or fat availability - called metabolic inflexibility - is associated with the development of muscle insulin resistance and type 2 diabetes^4^. These findings not only illustrate the importance of skeletal muscle in modulating blood glucose levels, but also indicate that in order to maintain metabolic health a fast switch between substrates is necessary to cope with the daily nutrient challenges.

To anticipate such metabolic adjustments at the cellular levels, peripheral tissues generate circadian oscillations by an intricate transcriptional-translational feedback loop, known as the molecular clock. This clockwork involves an activating limb, comprising the heterodimer of Brain and muscle ARNT-Like 1 (BMAL1) and circadian locomotor output cycles kaput (CLOCK). This complex promotes gene expression at E-boxes, and controls thereby the expression of period (PER), cryptochrome (CRY) and nuclear receptor subfamily 1, group D, member 1 (REVERBA). The repressing limb comprises REVERBA, REVERBB and the heterodimer of PER with CRY. The timely shifted translocation of the PER/CRY heterodimer into the nucleus inhibits the activating limb. Independently, REVERBA inhibits the expression of BMAL1^5^,^6^. In addition, the cellular energy status, reflected by nicotinamide adenine dinucleotide (NAD^+^), can activate sirtuin 1 (SIRT1), which in turn represses the active limb by deacetylation of both activating and repressing limb components 7. The fine-tuning of this mechanism is closely regulated on both limbs by post-transcriptional modifications^7^,^8^,^9^.

Apart from keeping time, this clockwork governs the transcription of clock-controlled genes, such as tissue-specific transcription factors of carbohydrate- and lipid metabolism^10^,^11^,^12^,^13^, but also of genes involved in mitochondrial biogenesis^13^,^14^. Notwithstanding, the peripheral molecular clock of skeletal muscle is also sensitive to hypothalamic-independent signals such as fasted-fed cycles and/or physical exercise^10^,^15^,^16^,^17^,^18^.

In recent years, evidence is emerging that disturbances in the circadian clock may lead to metabolic diseases. For example glucose homeostasis was severely disturbed in rodent knock out and deficiency models of BMAL1^19^,^20^,^21^,^22^, REVERBA^23^, PER^20^ and CRY^24^. Also liver- and overall body fat were adversely affected in knockout mice of BMAL1 and REVERBA. Lastly, the muscle-specific inactivation of Bmal1 in mice lead to muscle insulin resistance, altered muscle glucose metabolism and ultimately to substantially elevated muscle and body weight^25^.

Also in human studies, clock gene expression is associated with metabolic health, as adipose tissue clock gene expression was shown to correlate with waist circumference^26^ and BMI^27^. Furthermore, macronutrient availability has been shown to influence clock gene expression in blood monocytes of healthy subjects^28^. However, whether circadian rhythmicity of clock and metabolic genes in muscle is altered in humans with type 2 diabetes is so far unknown. In order to characterize the autonomous molecular clock of human muscle in relation to type 2 diabetes, a human primary myotube model was used. Human primary myotubes maintain many of the metabolic characteristics of their donor^29^,^30^,^31^. Moreover, it was recently shown that circadian rhythmicity of clock genes is maintained in human primary myotubes derived from healthy donors^32^. Therefore, human primary myotubes can be used to investigate if circadian rhythmicity in metabolic gene expression is affected in type 2 diabetes. Here, we cultured human primary myotubes from donors with a broad range in metabolic health status and insulin sensitivity, i.e. ranging from young healthy endurance trained athletes to type 2 diabetes patients. We aimed to investigate whether gene expression of key metabolic pathways in cultured human primary myotubes displayed circadian rhythmicity and whether this is impacted by the phenotypic characteristics of the donor.

## Results

This cell study examines circadian rhythmicity in human primary myotubes cultured from 4 phenotypically different donor groups: young endurance trained athletes (trained lean, TL), their age-matched young, lean sedentary controls (untrained lean, UL), healthy, obese subjects (OB) and patients with type 2 diabetes (T2D) that were matched for BMI and age with OB. Characteristics of the donors are provided in Table 1.

### Rhythmicity of genes encoding the core molecular clock and its repressor complex in cultured human primary myotubes is similar in metabolically distinct groups

**Figure 1:**
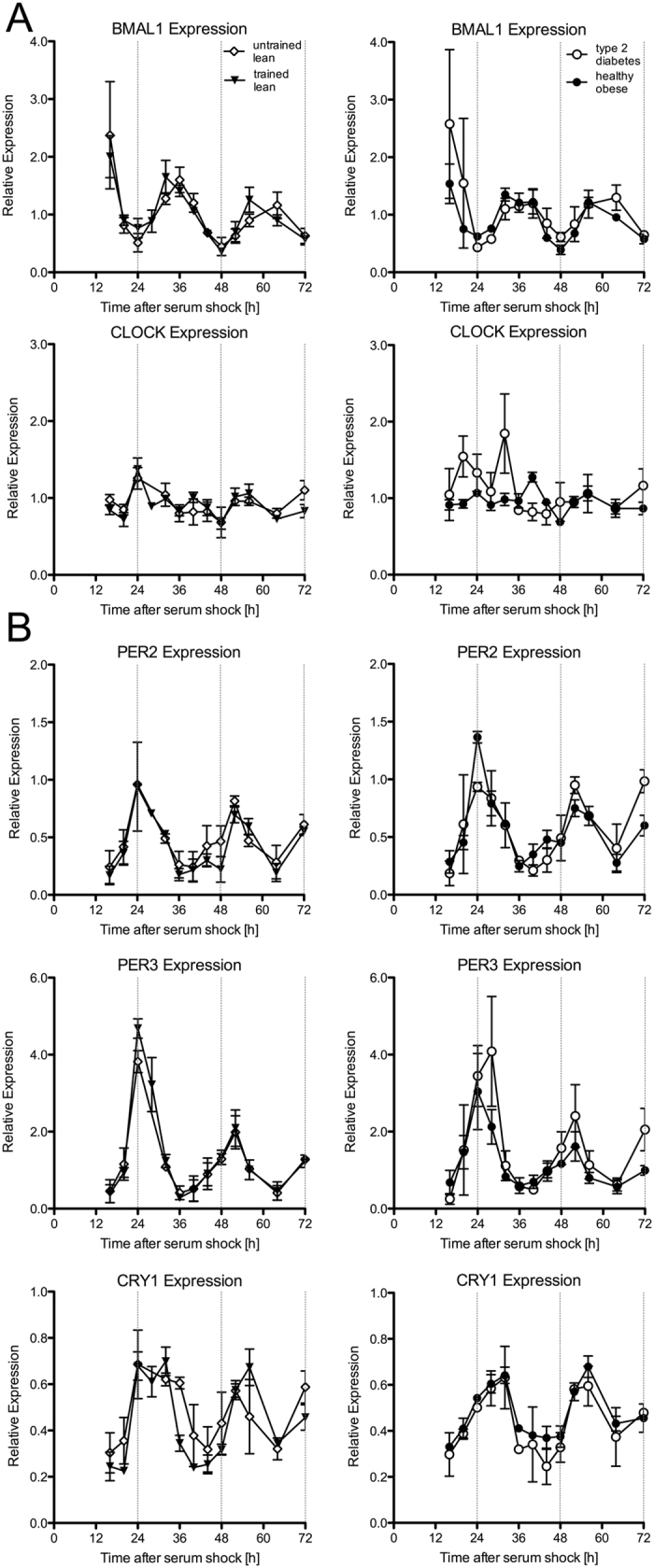
Rhythmic gene expression of core components of the molecular clock. The relative transcript abundance of the positive (**A**) and negative limb (**B**) components of the molecular clock was quantified by RT-PCR and normalized to the corresponding geometric mean of RPL26, CYPB and GUSB. Each value consists of the average of the independent cultures from three different donors. The value of the sample taken immediately after serum shock (0 h) was normalized to 1. Expression profiles of synchronized differentiated primary myotube cultures from trained lean (black triangle), untrained lean (open diamond), obese (black circle) and type 2 diabetes patients (open circle) are shown. Data are mean ± SEM.

We first determined gene expression profiles of the core molecular clock components to examine circadian rhythmicity. BMAL1 expression showed 24-hour rhythmicity in all four donor groups as detected by JTK_CYCLE (Fig. 1A, p < 0.01). One-way ANOVA analysis revealed that in all groups gene expression was indeed significantly different between time points (p < 0.05), and post-hoc analysis revealed significant differences between peaks at 36 h and 64 h after serum shock and a corresponding nadir at 24 h, 48 h and 72 h after serum shock in all groups (p < 0.05). Two-way ANOVA revealed no significant interaction effect for BMAL1 expression, suggesting that the rhythmicity of BMAL1 expression was not different between donor groups (p > 0.05).

It has previously been reported, that the heterodimer partner of BMAL1, CLOCK, features minimal circadian variation^33^,^34^. Here, we confirm that CLOCK gene expression revealed no circadian rhythmicity in human primary myotubes (Fig. 1A, JTK_CYCLE p > 0.05). The components of the repressor complex – PER1, PER2, PER 3 and CRY1, CRY2 – are known to oscillate in a 12 h to 16 h shift to the rhythm of BMAL1^32^,^35^. Indeed, PER2 and PER3 showed circadian rhythmicity for all donor groups (Fig. 1B, JTK_CYCLE p < 0.001). For PER2 one-way ANOVA analysis revealed that gene expression was indeed significantly different between time points of cells from untrained lean (UL) and sedentary obese (OB) (p < 0.05), and post-hoc analysis revealed significant differences in time with peaks at 24 h and 52 h and nadirs at 36 h and 64 h after serum shock, which is in anti-phase with the expression profile of BMAL1 (Fig. 1A,B). Although similar rhythmicity was visible in cells of trained lean (TL) and type 2 diabetic patients (T2D), differences in time did not reach statistical significance for PER2 (p = 0.13 and p = 0.12 in TL and T2D respectively) whereas one-way ANOVA revealed significant differences for all four donor groups for PER3.

Gene expression of CRY1 also showed significant circadian rhythmicity in all groups (Fig. 1B, JTK_CYCLE p < 0.01). In our hands, PER1 and CRY 2 were undetectably low in cultured human myotubes.

The circadian rhythm patterns of PER2, PER3 and CRY1 were not significantly different between donor groups, as we observed no interaction effect using two-way ANOVA analysis (p > 0.05).

**Figure 2:**
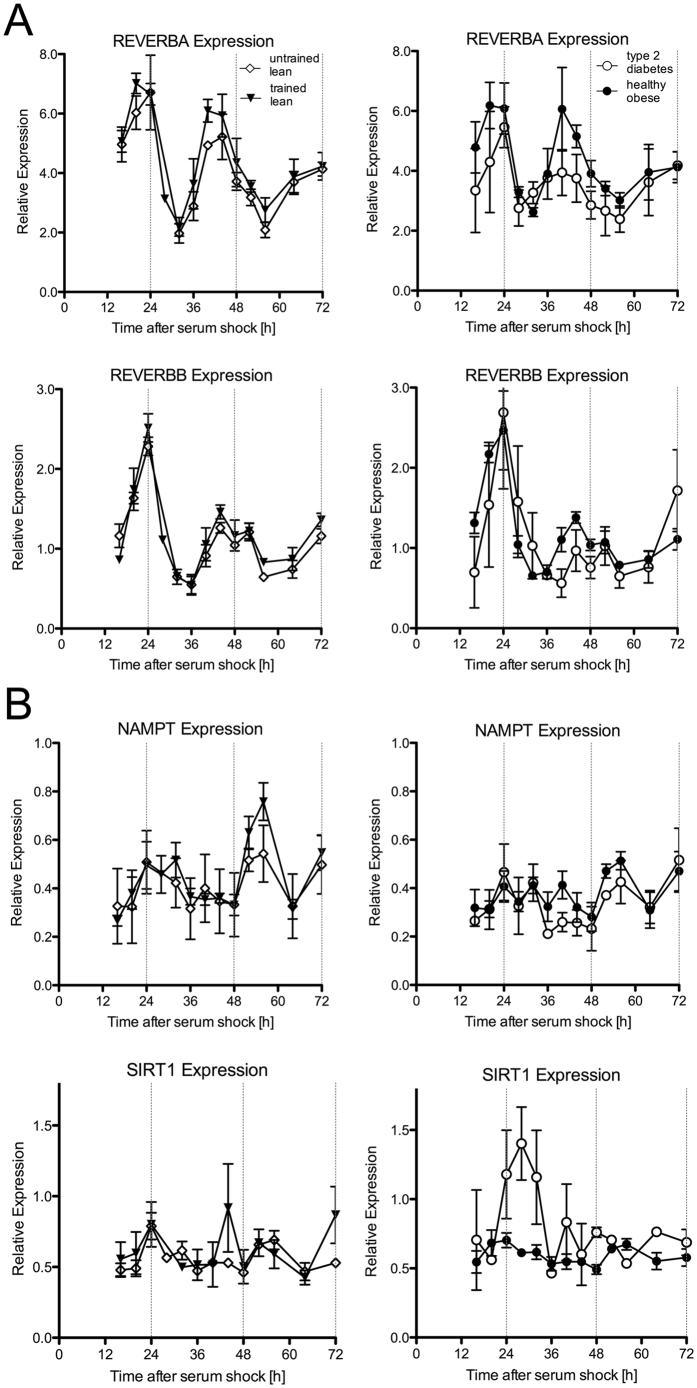
Rhythmic gene expression of molecular clock components. The relative abundance of transcripts that are under the control of the Ebox domain was quantified by RT-PCR and normalized to the corresponding geometric mean of RPL26, CYPB and GUSB. Each value consists of the average of the independent cultures from three different donors. The value of the sample taken immediately after serum shock (0 h) was normalized to 1. Expression profiles of synchronized differentiated primary myotube cultures from trained lean (black triangle), untrained lean (open diamond), obese (black circle) and type 2 diabetes patients (open circle) are shown. Data are mean ± SEM.

Next, we measured the transcripts of genes that have been reported to be under the transcriptional control of CLOCK and BMAL1, and to be involved in the fine-tuning of the circadian clock. Both for REVERBA and REVERBB, 24-hour circadian rhythmicity was observed in athletes, lean sedentary and obese subjects (Fig. 2A, JTK_CYCLE p < 0.001), but the rhythm was dampened in type 2 diabetes patients (Fig. 2A, JTK_CYCLE p > 0.05). One-way ANOVA analysis revealed significantly different REVERBA levels between time points in all groups (Fig. 2A, p < 0.05). Post-hoc analysis revealed that REVERBA peaked at 20 to 24 h and again at 40 h to 44 h, with the corresponding nadirs at 32 h and 52–56 h. For REVERBB, highest expression was determined at 24 h and at 48 h, and lowest expression at 16 h, 36 h and 56 h (Fig. 2A) in all groups except for type 2 diabetes patients (ANOVA TL, UL, OB: p < 0.001, T2D: p = 0.473).

A circadian rhythm was observed for SIRT1 only in athletes (Fig. 2B, p < 0.05). One-way ANOVA also revealed a statistical significant time effect in athletes only (p < 0.05), with highest expression at 24 h and 48 h, and lowest expression at 36 and 64 h. In concordance with SIRT1 expression, analysis of NAMPT expression also confirmed a circadian rhythm in athletes only (Fig. 2B, p < 0.001), although one-way ANOVA did not reveal significant differences between the time points.

### Modest circadian rhythmicity in expression of genes involved in substrate metabolism

**Figure 3:**
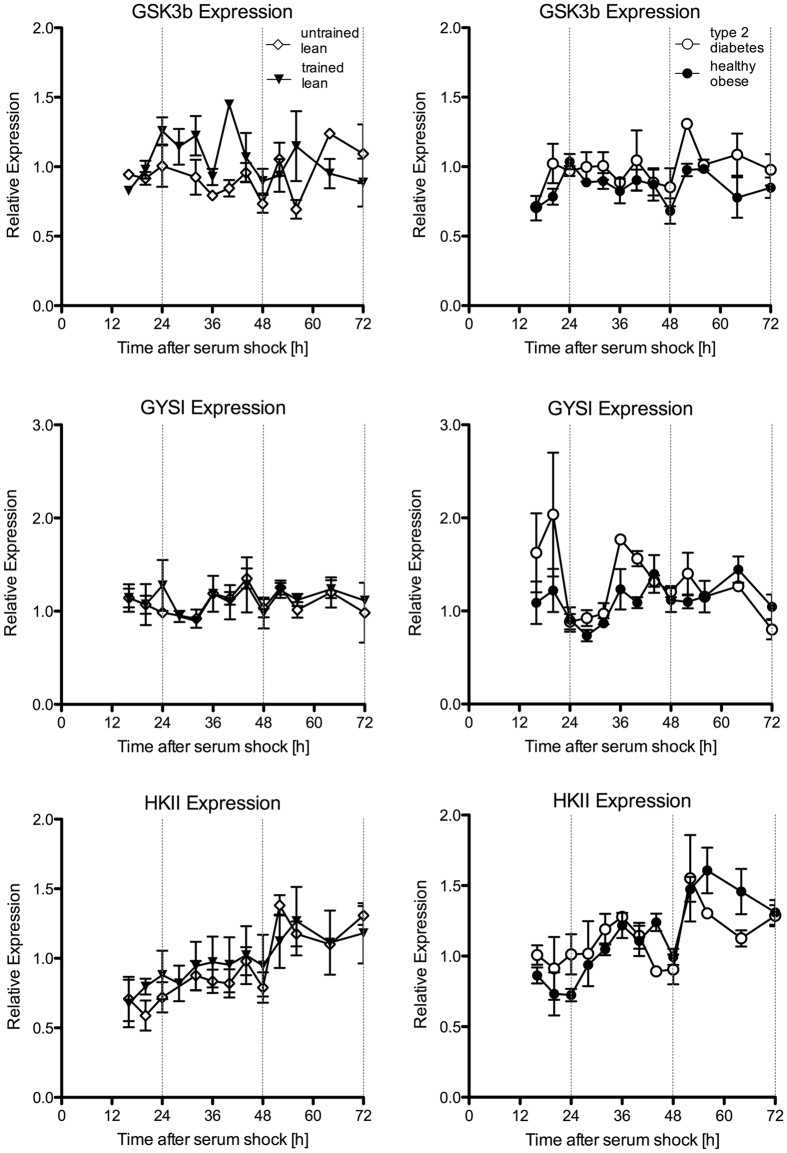
Expression profiles of cellular glucose metabolism genes. The relative abundance of transcripts that encode proteins of cellular glucose and glycogen metabolism was quantified by RT-PCR and normalized to the corresponding geometric mean of RPL26, CYPB and GUSB. Each value consists of the average of the independent cultures from three different donors. The value of the sample taken immediately after serum shock (0 h) was normalized to 1. Expression profiles of synchronized differentiated primary myotube cultures from trained lean (black triangle), untrained lean (open diamond), obese (black circle) and type 2 diabetes patients (open circle) are shown. Data are mean ± SEM.

We next measured transcript abundance of genes that are key regulators in cellular glucose metabolism. Circadian rhythmicity determined by JTK_CYCLE was found in gene expression of glycogen synthase 1 (GYS1) for type 2 diabetes patients only, but not for its inhibitory regulator – the glycogen synthase kinase 3 beta (GSK3b). For hexokinase II (HKII) JTK_CYCLE indicated rhythmicity in sedentary lean and tendencies in obese and type 2 diabetic donors (Fig. 3, UL p < 0.05; OB p = 0.07; T2D p = 0.09, TL p = 1.00); However, one-way ANOVA revealed no significant differences in HKII gene expression in time in sedentary lean (p > 0.05). Also, as can be seen from Fig. 3, the pattern of HKII suggests a linear increase in HKII over time. Finally, we measured insulin receptor substrate 1 (IRS1) – a gene involved in insulin-stimulated glucose uptake – which only revealed a rhythmic pattern using JTK_CYCLE in athletes (Supplemental Figure 3, JTK_CYCLE p < 0.01).

**Figure 4:**
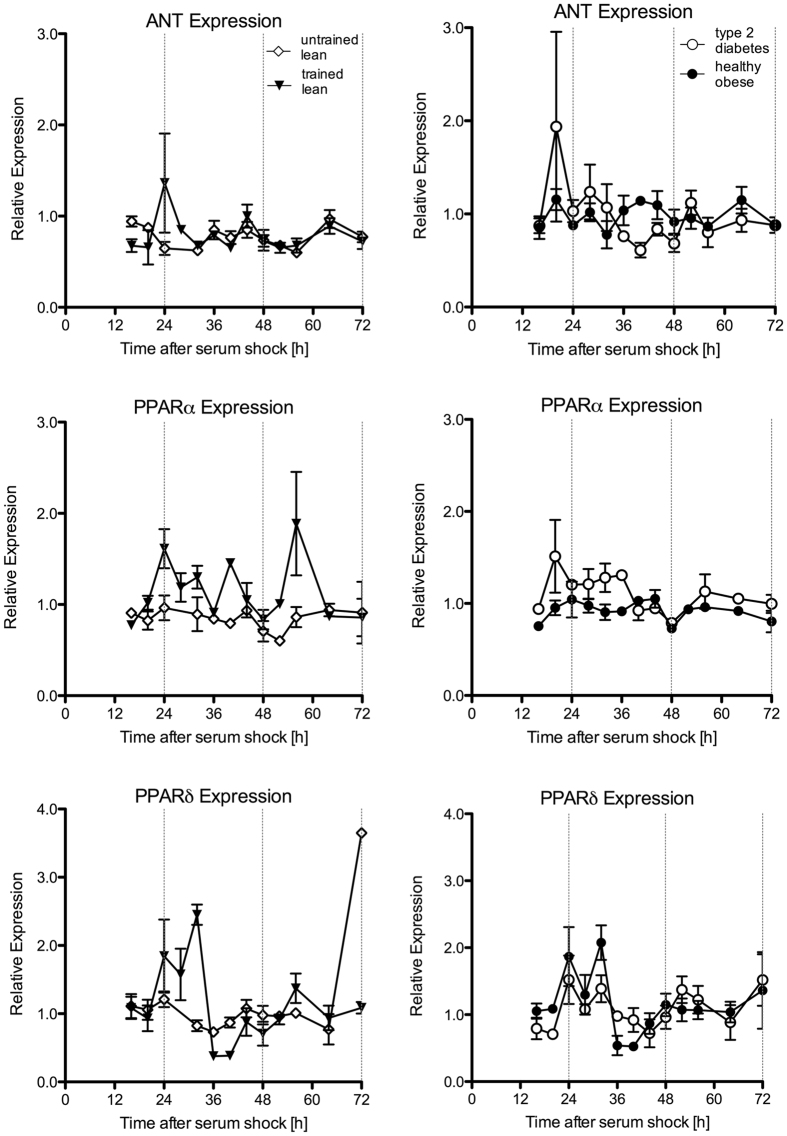
Expression profiles of genes involved in mitochondrial metabolism. The relative transcript abundance of transcripts that encode proteins involved in mitochondrial function and biogenesis was quantified by RT-PCR and normalized to the corresponding geometric mean of RPL26, CYPB and GUSB. Each value consists of the average of the independent cultures from three different donors. The value of the sample taken immediately after serum shock (0 h) was normalized to 1. Expression profiles of synchronized differentiated primary myotube cultures are plotted as follows: trained lean (black triangle) against untrained lean (open diamond) and obese (black circle) against type 2 diabetics (open circle). Data are mean ± SEM.

We next also determined gene expression of several genes in mitochondrial function. Therefore, we measured the mitochondrial adenine nucleotide transporter (ANT) that exchanges ATP and ADP in the mitochondrial matrix. Further, we quantified the peroxisome proliferator-activated receptor alpha (PPARα) and delta (PPARδ), which both are involved in regulating lipid metabolism and mitochondrial biogenesis. Circadian rhythmicity was found in gene expression of ANT in sedentary lean and for PPARα in athletes (JTK_CYCLE p < 0.05), but one-way ANOVA did not reveal significant differences in time for any of the groups (Fig. 4, p > 0.05). For PPARδ, circadian rhythmicity was observed in athletes, obese and T2D using JTK-cycle (JTK_CYCLE p < 0.05), and one-way ANOVA analysis revealed significantly different gene expression levels of PPARδ between time points in athletes and obese, p < 0.001 and p < 0.009, respectively. Post-hoc analysis revealed that PPARδ was significantly higher at 28 h vs. 36 h in TL. Furthermore, we determined gene expression of DGAT1, the rate-limiting enzyme in the conversion of diacylglycerol into triacylglycerol. Circadian rhythm was revealed in lean sedentary subjects only using JTK-CYCLE (Supplemental Figure 3, p > 0.05), but one-way ANOVA did not reveal significant differences in time in lean sedentary subjects (Supplemental Figure 3).

### Linking circadian rhythmicity with the metabolic phenotype of the donor

**Figure 5:**
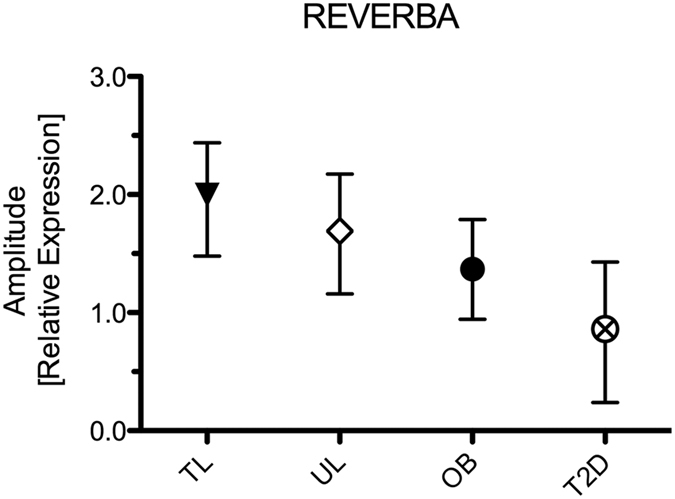
Correlative analysis of circadian rhythmicity with the metabolic phenotype of the donor. The plot displays the association (Spearman’s Rho test for nominal data) between the metabolic phenotype of the donor with the amplitude of the JTK_CYCLE based peak and nadir for REVERBA. Data are relative expression amplitude with 95% CI as error bars.

The donors of the biopsies that were cultured for this study were grouped phenotypically to represent a group of young endurance trained athletes (TL, n = 3), aged-matched, lean sedentary controls (UL, n = 3), normoglycemic middle-aged obese subjects (OB, n = 3) and BMI and age-matched (with OB) patients with type 2 diabetes (T2D, n = 3). In these groups, insulin sensitivity (measured as M-value) progressively deteriorated from TL > UL > OB > T2D (Table 1). To link putative differences in rhythmicity with the metabolic phenotype of the donor, we applied Spearman Rho’s correlation analysis for nominal parameters and observed that with progressive disease state the amplitude of the gene-expression profile of REVERBA (rs = −1.0, p < 0.05) also declined progressively (Fig. 5). For none of the other genes we measured, such a correlation was observed.

## Discussion

Rodent studies have revealed the importance of the CLOCK/BMAL1 component in the regulation of autonomous circadian rhythm in skeletal muscle^14^,^25^. Recently, circadian rhythmicity was also shown in human primary myotubes^32^, which we confirm here. Our results reveal that circadian rhythmicity for the core clock components was not affected by the metabolic status of the donor. Interestingly, in comparison to cultures of healthy donors REVERBA and REVERBB analysis showed no circadian rhythmicity in human primary myotubes derived from type 2 diabetes patients, and rhythmicity for SIRT1 and NAMPT was only observed in myotubes from endurance trained athletes. For REVERBA we observed that with donor groups of progressively lower insulin sensitivity (TL > UL > OB > T2D), the amplitude of the rhythm progressively declined. These findings may suggest that the molecular clock system in human skeletal muscle is affected by metabolic condition and may be involved in the aetiology of chronic metabolic disease.

The aim of the current study was to examine if circadian rhythmicity in skeletal muscle is affected by the characteristics of the donors, whom were ranging in metabolic health from endurance trained athletes to metabolically-compromised T2D patients. We find that serum shock is capable of inducing synchronization in human primary myotubes from a skeletal muscle biopsy. In line with the study of Balsalobre *et al.*^36^, oscillation of autonomous clock components persists at least throughout 72 h after serum shock synchronization. This robust oscillation was reflected by all measured components of the transcriptional-translational feedback loop. Especially, BMAL1 and REVERBA demonstrated the highest amplitudes in rhythmicity. Furthermore, the characteristic phase delay of REVERBA/B, PER and CRY to BMAL1, previously observed in human biopsies^34^,^35^ and human primary myotubes^32^, was confirmed in the present study.

It has previously been demonstrated that cultured myotubes retain their *in vivo* muscle phenotype in terms of insulin sensitivity^29^,^30^ and mitochondrial lipid metabolism^31^. Even though it is not uncommon for genes to possess lower expression in *in vitro* models than in muscle biopsies, we previously did not observe this in our myotubes^37^. This, along with maintenance of the *in vivo* phenotype in culture, indicates that cultured myotubes from phenotypically characterised donors appears a valid and valuable model in our hands. In the current study, we confirm that also after the procedure of serum shock, mitochondrial function was reduced in type 2 diabetic patients compared to obese controls (Supplemental Figure 2). Despite this, we did not find any differences in circadian rhythmicity in the core molecular clock components BMAL1, CRY1, PER2 and PER3 between donor groups, which could indicate that the core molecular clock is not affected in muscle of type 2 diabetic patients, as was shown in temporal series of human white adipose tissue biopsies^35^. However, circadian rhythmicity was affected by the donor characteristics for REVERBA/B and for SIRT1 and NAMPT. Thus, circadian rhythmicity for REVERBA/B was detected in all groups except for type 2 diabetes patients. Furthermore, interestingly, we could only confirm rhythmicity for NAMPT and SIRT1 expression in human primary myotubes derived from endurance-trained athletes. As SIRT1 and NAMPT have been shown to be associated with metabolic health^38^,^39^,^40^ it is interesting that intrinsic rhythmicity is less robust in sedentary lean, obese and T2D donor cells. Furthermore, it should be noted that all cells were cultured under optimized and constant cell culture conditions. Given the differences in metabolic flexibility between diabetic patients and (un)trained healthy lean subjects, it may be that the differences in circadian rhythmicity as measured in the current model may be underestimated. In that context, it may be interesting to expose synchronized skeletal myotubes with excess palmitate or glucose substrates and investigate whether circadian rhythmicity is more pronouncedly affected under such diabetogenic conditions.

The link between the molecular clock and metabolic homeostasis has previously been examined with a focus on insulin sensitivity. In mice e.g. it has been shown that rhythmic insulin action^41^ depends on the clock gene BMAL1^25^,^41^. In humans, glucose tolerance was shown to display a diurnal pattern^42^,^43^ and forced desynchronization has been reported to induce pre-diabetic conditions in healthy young men^44^. This may be explained by rhythmic insulin secretion^45^, but also by rhythmic action of insulin^41^,^46^ in target organs like skeletal muscle. In the present study, we show rhythmicity of regulatory genes in glucose and lipid metabolism or mitochondrial biogenesis using JTK_CYCLE, but these rhythms were not as robust as has been reported for murine liver^7^,^47^,^48^ heart or skeletal muscle^49^. Further, murine skeletal muscle was shown to maintain rhythms of glucose metabolites and corresponding rate-limiting enzymes, such as GLUT4, HKII and PDK4^25^. This contrasts with our study in synchronized human myotubes in which we did not observe rhythmic expression of HKII, GYSI or GSK3B mRNA in all groups. In addition, no circadian expression of IRS1 was detected, in contrast to murine muscle and liver^50^. Whilst lipid metabolism is highly diurnal in mouse liver, white adipose tissue and aorta, only moderate rhythm was detected in the present study for DGAT1^10^,^51^ and PPARα^10^,^12^ or PPARδ^11^. In line with the absence of oscillations in metabolic gene expression, the mitochondrial gene ANT is expressed with minimal variation over time. It should be noted that even when rhythmicity in metabolic gene expression was observed, the amplitude of the rhythm was very low in comparison to the clock genes.

### Limitations of the present study

Although the present study is the first to present data in differentiated cultured human myotubes of 12 different donors ranging vastly in metabolic health and with a relatively high time resolution (13 RNA sampling points within 72 hours), a couple of limitations deserve to be mentioned. First we did not quantify sleep quality, nor did we assess the circadian phase of the donor at the time of the biopsy. Hence, we cannot exclude a putative carry over effect of any abnormalities in sleep or rhythmicity to the cells in culture. We did, however, take all muscle biopsies between 8.00 and 9.00 PM from patients without recognized sleep abnormalities. Second, we assessed metabolic data just prior to taking the biopsy and did not follow-up the putative rhythmic profile of metabolic data of the donors for 72 hours, which would have permitted a direct comparison between *in vivo* data on rhythmicity with rhythmicity in the *ex vivo* cell model. Finally, one should note that the data presented are derived from cells cultured from 3 different donors per group. Although we feel that the fact that these are human donors, whom are metabolically well characterised, clearly is a strength of the present study and most likely better resembles the human situation than immortalized rodent muscle cell lines like C2C12 or L6 myotubes, one should note that it is premature to state that data from these 3 donors per group are representative for the total population of people who qualify as trained lean, untrained lean, obese or type 2 diabetic.

In conclusion, we demonstrate, that circadian components of the molecular clock, namely REVERBA/B, SIRT1 and NAMPT, that are associated with mitochondrial function and metabolic health, showed lack of circadian rhythmicity in human primary myotubes from type 2 diabetic patients. Modest circadian rhythmicity was observed in few downstream genes of the molecular clock system, such as metabolic genes involved in glucose and lipid metabolism. Future studies are needed to examine if circadian rhythmicity can also be observed in physiological measures such as insulin sensitivity or mitochondrial function in cultured human muscle cells of insulin resistant subjects.

## Methods

### Study design and subject characteristics

In the present study, we included human primary myotubes from subjects that had previously participated in our studies^37^,^52^. We cultured human primary myotubes from the following four groups: (1) young endurance trained athletes (TL) and (2) their age-matched young, lean sedentary controls (UL) and (3) healthy, obese subjects (OB) and (4) BMI- and age-matched type 2 diabetic patients (T2D) (Table 1). Inclusion criteria for endurance trained athletes were performing endurance exercise (long-distance running, cycling, swimming) at least three times a week for the past 2 years and a VO2 max >55 ml/min/kg; all other subjects performed less than 1 h of exercise per week for the past 2 years. Type 2 diabetic patients were diagnosed at least 1 year prior to the study, were non-insulin dependent, were well-controlled (HbA1c <64 mmol/mol), and had no diabetes-related comorbidities. All type 2 diabetes patients had been treated with metformin or metformin + sulfonylureas for at least 6 months. All studies were approved by the local Medical Ethical Committee of Maastricht University according to the declaration of Helsinki principles of 1975, as revised in 1983. All participants gave written informed consent.

### Hyperinsulinemic-euglycemic clamp

Peripheral insulin sensitivity was measured by a two-step hyperinsulinemic-euglycemic clamp, according to Sparks *et al.*^37^. The M-value was calculated as the glucose infusion rate required to maintain euglycemia (5.5 mmol/l) under hyperinsulinemic conditions (insulin infusion at 40 mU/m^2^/min) and was corrected for fat-free mass (FFM).

### Maximal aerobic exercise

A stepwise progressive exercise test until exhaustion was performed on a stationary bike to measure whole body oxygen uptake as a reflection of physical fitness. Simultaneous breath gas analysis to measure maximal oxygen (VO_2_ max) uptake and carbon oxide production (VCO_2_) was performed under standardized laboratory conditions. Exercise was stopped when the subjects were unable to maintain cadence >60 RPM, heart rate exceeded 220 minus age or at a respiratory exchange ratio >1.10.

### Primary muscle cell cultures

As described before^37^, muscle biopsy specimens were taken from the musculus vastus lateralis according to Bergström^53^ and processed the same day for cell culture. All biopsies were obtained after a good night of sleep in the overnight fasted state between 8 to 9 AM. Self-reported day-night rhythm and sleep quality was good for all subjects. Primary skeletal muscle cell cultures were established by isolating and growing needle biopsy derived satellite cells in media supplemented with 16% FBS (Gibco, Thermo Fisher Scientific, Waltham, USA) at 37 °C and 5% CO_2_. Differentiation to multi-nucleated skeletal myotubes was induced by seeding mono-nucleated satellite cells confluently in differentiation media (passage  < 5); Minimum Essential Medium α (Gibco, Thermo Fisher Scientific, Waltham, USA) supplemented with 2% FBS (Gibco, Thermo Fisher Scientific, Waltham, USA) and fetuin (Sigma-Aldrich, St. Louis, USA). Every other day differentiation media was changed.

To examine if key characteristic defects in muscle of patients with type 2 diabetes (mitochondrial dysfunction and compromised glucose handling at the post-insulin receptor level observed in the type 2 diabetic state^54^) are also present in cultured myotubes, we examined mitochondrial oxygen consumption rate by Seahorse and basal gene expression of glycogen synthase. In line with previous *in vivo* observations we observed compromised mitochondrial function in T2D, measured as lower basal as well as maximal mitochondrial oxygen consumption rate in T2D than in OB (Supplemental material Figure 2). Moreover, we observed that expression of the gene encoding for the rate limiting step in glycogen synthesis (GYS1) was some 50% lower (p = 0.031) in myotubes cultured from patients with T2D than in myotubes from the obese.

These findings, along with our previously reported observation that abrogated myocellular lipid handling in patients with T2D was retained in myotubes cultured form these donors^37^ indicates that these cultured myotubes are a valid model to represent the different metabolic phenotypes of their respective donors and hence are a good cell model for *in vitro* studies towards the myocellular molecular clock.

### *In-vitro* synchronization of primary myotubes

On day seven of differentiation, multi-nucleated skeletal myotubes were synchronized by serum shock^36^. Serum shock consisted of a two-hour incubation with fresh differentiation media, supplemented with 50% horse serum (Gibco, Thermo Fisher Scientific, Waltham, USA). After serum shock, cultures were washed and supplemented with fresh differentiation media. All individual cultures of the four donor groups were synchronized on the same day. Cultures were harvested 0, 16, 20, 24, 28, 32, 36, 40, 44, 48, 52, 56, 64, or 72 hours after synchronization by placing the culture plate on ice and washing thoroughly with Dulbecco’s Phosphate-Buffered Saline (Gibco, Thermo Fisher Scientific, Waltham, USA). Supernatant was immediately removed and cell lysis was initiated.

### Transcript quantification and normalization

RNA isolation was performed on-dish by TRIzol lysis (Qiagen, Hilden, Germany). RNA was further purified by the RNeasy kit from Qiagen (Hilden, Germany). The quality and yield of RNA was assessed using a NanoDrop spectrophotometer (Thermo Fisher Scientific, Waltham, USA). The high-capacity RNA-to-cDNA kit from Applied Biosystems (Foster City, USA) was used for transcribing 0.5 μg RNA to cDNA. Transcript abundance was determined using a 7900HT Fast Real-Time PCR System (Applied Biosystems, Waltham, USA). Three housekeeping genes (HKG), namely RPL26, GUSB and CYPB, were identified to be stably expressed in synchronized multi-nucleated myotubes throughout time (Supplemental Figure 1) by both GeneNorm^55^ and Bestkeeper^56^. To further minimalize the variability in housekeeping gene normalization, the geometric mean of the three housekeeping genes, which showed strong robustness in time, was used. Therefore, the geometric mean was used as the internal reference for the comparative gene expression analysis in the remainder of the study. Thus, each sample was normalized to the corresponding sample at time of serum shock by comparative gene expression analysis.

### Mitochondrial function – Oxygen Consumption Rate (OCR) measurements

To determine whether cultures of the four donor groups kept the donor phenotype, human primary myotube were plated and differentiated on a Seahorse Bioscience 96-well plate. One hour prior to measurement, differentiation medium was changed to Seahorse Biosciences XF media according to the XF Cell Mito Stress Test Kit (Seahorse Bioscience, USA, North Billerica). Oligomycin, FCCP and rotenone with antimycin A was added to the cells to determine the oxygen consumption rates for basal mitochondrial respiration, maximal respiration and mitochondrial proton leak (Supplemental Figure 2). OCR was corrected for non-mitochondrial respiration (rotenone with antimycin A respiration).

### Statistical analysis

Results are presented as means ± SEM derived from three independent cultures of each donor group. Gene expression was tested for rhythmicity using the JTK_CYCLE package for R in Windows 8^57^; for this, data from 4-hourly sampled time points (between 16 h and 56 h) determining the best fit for 20, 24 or 28 hours of period length was used. In case JTK_CYCLE indicated rhythmicity, we tested whether gene expression between time points 16 h to 56 h was statistically significant by one-way ANOVA, followed by Bonferroni post-hoc analysis for determination of peak and nadir in rhythmic expression profiles. If significant rhythmicity was observed, two-way ANOVA was also performed to detect group differences in circadian rhythmicity between groups. A p-value < 0.05 was considered statistically significant. Spearman’s Rho correlative analyses for nominal data were performed to explore correlations between the amplitude of rhythmic genes with the donor group. Statistical analyses were performed using the statistical program SPSS 23.0 for Mac OS X.

## Additional Information

**How to cite this article**: Hansen, J. *et al.* Synchronized human skeletal myotubes of lean, obese and type 2 diabetic patients maintain circadian oscillation of clock genes. *Sci. Rep.* **6**, 35047; doi: 10.1038/srep35047 (2016).

## Supplementary Material

Supplementary Information

## Figures and Tables

**Table 1:** Donor Characteristics.

		Trained Lean			Untrained Lean			Obese			Type 2 Diabetic		
		Average	±SEM	N	Average	±SEM	N	Average	±SEM	N	Average	±SEM	N
Age	[yr]	22.0	1.0	3	22.3	1.2	3	57.3	12.9	3	59.0	7.0	3
BMI	[kg/m^2^]	19.2	1.4	3	21.3	1.4	3	30.2	1.4	3	30.3	0.3	3
M-Value 40 mU	[mmol/kg_FFM_/min]	76.1	5.9	3	50.6	13.6	3	26.8	3.0	3	19.5	10.8	3
Fat mass	[%]	13.8	0.3	3	15.5	5.4	3	30.9	4.9	3	34.7	3.4	3
VO2-max	[mL/min/kg]	61.4	5.1	3	41.8	0.8	3	28.4	3.7	3	27.5	4.3	3

Data are expressed as means ± standard error of mean.
