# Supplementary material for: Synchronized human skeletal myotubes of lean, obese and type 2 diabetic patients maintain circadian oscillation of clock genes
Source: Sci Rep. 2016 Oct 19;6:35047. doi: 10.1038/srep35047 (PMC5069469; doi:10.1038/srep35047)
Supplement: Supplementary Information [file srep35047-s1.pdf]

Supplementary Information

Synchronized human skeletal myotubes of lean, obese and type 2 diabetic patients maintain circadian oscillation of clock genes

Jan Hansen[1], Silvie Timmers[1], Esther Moonen-Kornips[1], Helene Duez[2,3,4], Bart Staels[2,3,4], Matthijs KC Hesselink[1], Patrick Schrauwen[*,1]

[1]Department of Human Biology and Human Movement Sciences, NUTRIM School for Nutrition and Translational Research in Metabolism, Maastricht University Medical Center, Maastricht, The Netherlands.
[2]Institut Pasteur de Lille, Lille, France.
[3]Institut National de la Santé et de la Recherche Médicale Unité Mixte de Recherche 1011, Lille, France.
[4]Université Lille, European Genomic Institute for Diabetes, Lille, France.

Content

Supplementary Figure 1: Temporal gene expression stability of house keeping genes

p.1

Supplementary Figure 2: Respiratory capacity of donor phenotypes in cultured myotubes

p.2

Supplementary Figure 3: Expression profiles of IRS1 and DGAT1

p.3

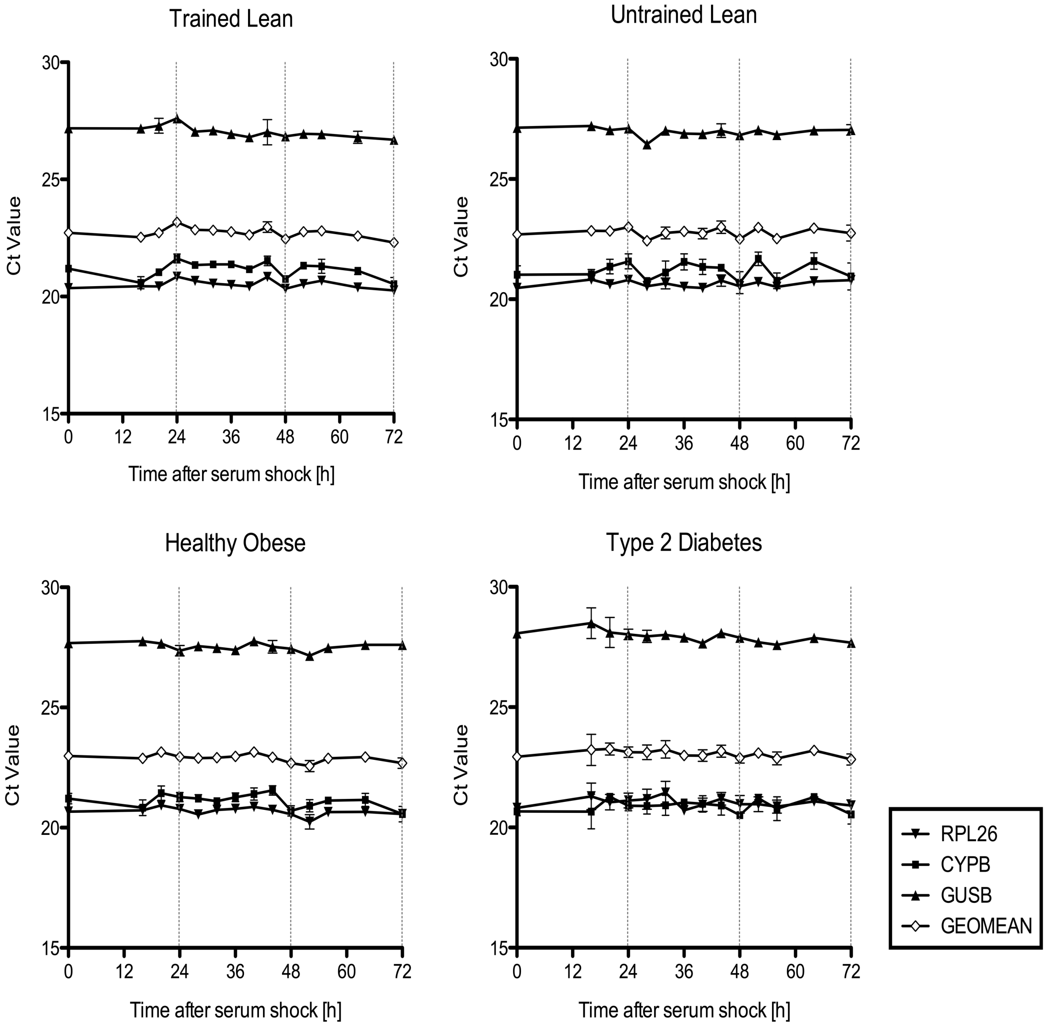

S.Figure. 1: Temporal gene expression stability of house keeping genes

Gene Expression of the housekeeping genes RPL26 (reverse black triangles), GUSB (black triangle) and CYPB (black squares) in time after serum shock (hours). The geometric mean (open diamonds) consists of the raw ct-values of all three housekeeping genes per time point. Data are mean ± SEM. n = 3 for each group.

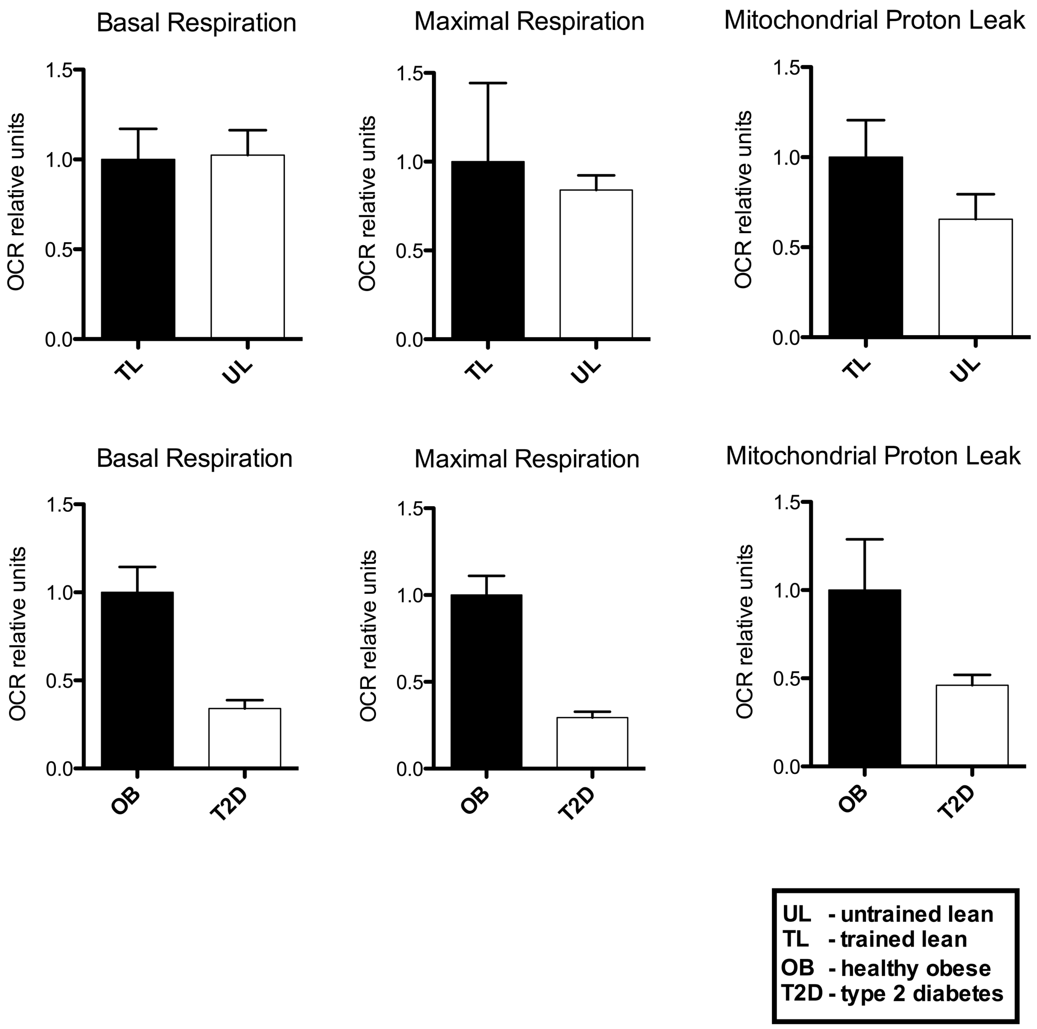

S.Figure 2: Respiratory capacity of donor phenotypes in cultured myotubes

The oxygen consumption rate (OCR) of the mitochondrial basal respiration (A), maximal respiration (B) and mitochondrial proton leak (C) are depicted relative to the control group (TL vs UL; OB vs T2D). OCR is corrected for non-mitochondrial respiration (antimycin A and rotenone). Data are mean ± SEM.

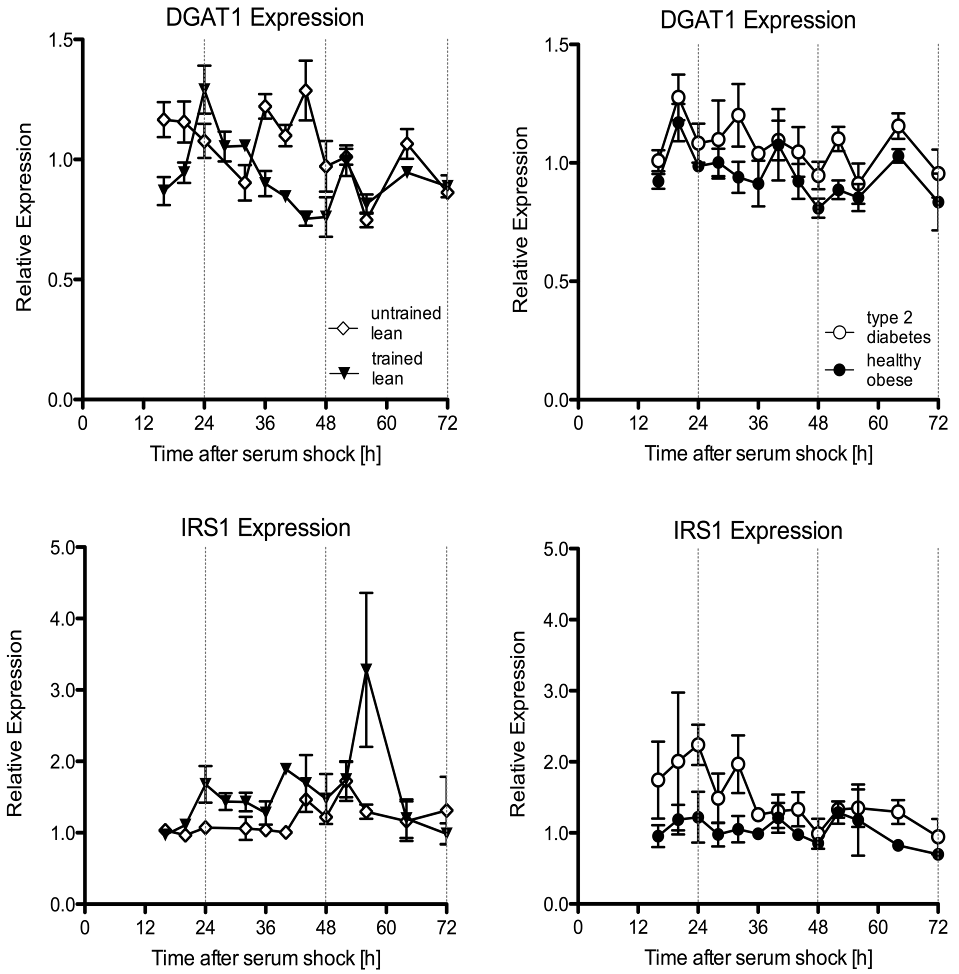

S.Figure 3: Expression profiles of IRS1 and DGAT1

The relative transcript abundance of IRS1 that encodes a protein involved in intracellular insulin signaling and DGAT1 that encodes a protein involved in triglyceride synthesis was quantified by RT-PCR and normalized to the corresponding geometric mean of RPL26, CYPB and GUSB. Each value consists of the average of the independent cultures from three different donors. The value of the sample taken immediately after serum shock (0h) was normalized to 1. Expression profiles of synchronized differentiated primary myotube cultures are plotted as follows: trained lean (black triangle) against untrained lean (open diamond) and obese (black circle) against type 2 diabetics (open circle). Data are mean ± SEM
